# Knowledge and appropriateness of care of family physicians and physiotherapists in the management of shoulder pain: a survey study in the province of Quebec, Canada

**DOI:** 10.1186/s12875-023-01999-6

**Published:** 2023-02-16

**Authors:** Véronique Lowry, Patrick Lavigne, Diana Zidarov, Kadija Perreault, Jean-Sébastien Roy, François Desmeules

**Affiliations:** 1 School of Rehabilitation, Faculty of Medicine, University of Montreal, Montreal, QC Canada; 2 Orthopaedic Clinical Research Unit, Maisonneuve-Rosemont Hospital Research Center, Montreal, QC Canada; 3 Department of Surgery, Faculty of Medicine, University of Montreal, Montreal, QC Canada; 4 Centre de recherche interdisciplinaire en réadaptation (CRIR), Montreal, QC Canada; 5 Institut universitaire sur la réadaptation en déficience physique de Montréal (IURDPM), Montréal, Québec Canada; 6 Department of Rehabilitation, Faculty of Medicine, Laval University, Quebec City, QC Canada; 7 Centre interdisciplinaire de recherche en réadaptation et intégration sociale (Cirris), Quebec City, QC Canada

**Keywords:** Shoulder, Survey, Clinical Practice Guidelines, Evidence-based practice, Family Physicians, Physiotherapists, Primary Care, Diagnosis, Management, Treatment

## Abstract

**Background:**

Shoulder pain is difficult to diagnose and treat with half of those affected still symptomatic six months after initial consultation. This may be explained by primary care management not conforming to evidence-based practice. This survey evaluated physiotherapists (PTs) and family physicians' (FPs) knowledge and appropriateness of care in shoulder pain management.

**Methods:**

A survey sent to PTs and FPs in the province of Quebec, Canada presented four clinical vignettes with cases of rotator cuff (RC) tendinopathy, acute full-thickness RC tear, adhesive capsulitis and traumatic anterior glenohumeral instability. Respondents indicated diagnosis, indications for imaging, specialists’ referrals, and choice of treatments. Answers were compared to recommendations from clinical practice guidelines (CPGs). Participants’ responses were compared between types of providers with Fisher’s exact test.

**Results:**

Respondents (PTs = 175, FPs = 76) were mostly women with less than ten years of experience. More than 80% of PTs and 84% of FPs correctly diagnosed cases presented. Despite this practice not being recommended, more FPs than PTs recommended an imaging test in the initial management of RC tendinopathy (30% compared to 13%, *p* = 0.001) and adhesive capsulitis (51% compared to 22%, *p* = 0.02). For full-thickness RC tear and shoulder instability, up to 72% of FPs and 67% of PTs did not refer to a specialist for a surgical opinion, although recommended by CPGs. For RC tendinopathy, 26% of FPs and 2% of PTs (*p* < 0.001) would have prescribed a corticosteroid infiltration, which is not recommended in the initial management of this disorder. For adhesive capsulitis, significantly more FPs (76%) than PTs (62%) (*p* < 0.001) suggested an intra-articular corticosteroid infiltration, as recommended by CPGs. For all presented vignettes, up to 95% of family physicians adequately indicated they would refer patients for physiotherapy. In prioritizing rehabilitation interventions, up to 42% of PTs did not consider active exercises as a priority and up to 65% selected passive modalities that are not recommended for all shoulder pain vignettes.

**Conclusions:**

Most FPs and PTs were able to make adequate diagnoses and select appropriate treatments for shoulder pain, but practices opposed to evidence-based recommendations were chosen by several respondents. Further training of FPs and PTs may be needed to optimize primary care management of different shoulder disorders.

**Supplementary Information:**

The online version contains supplementary material available at 10.1186/s12875-023-01999-6.

## Background

Shoulder pain affects up to two out of three people in a lifetime and is a leading cause of disability in the adult population [1]. The high level of disability and chronicity experienced by individuals with shoulder pain as well as its burden on the healthcare system and society may in part be explained by suboptimal primary care management [2–4]. Patients with shoulder pain usually consult their family physician [5], but their diagnosis often relies on the unnecessary use of expensive diagnostic imaging tests [6] that can induce delays in treatment, increase costs and lead to overdiagnosis and overtreatment [7]. The primary care management of shoulder pain often includes references to medical musculoskeletal (MSK) specialists such as orthopedic surgeons, even though most patients’ conditions do not require surgery [8].

Physiotherapists are specialists in MSK disorders with expertise in education and exercise interventions to effectively treat shoulder pain [9, 10]. In Canada, physiotherapists are considered primary care providers since patients can access physiotherapy services directly, without a referral [11]. Despite their knowledge in managing MSK disorders reported as being higher than that of family physicians or other physicians not specialized in MSK care [12], a recent systematic review reported that physiotherapists may use low value modalities that are not recommended in the management of shoulder pain [4].

Several high-quality CPGs with recommendations related to diagnosis as well as conservative and surgical management of shoulder pain have been developed and published in the past years [6, 13–18]. To improve primary care offered by physiotherapists and family physicians, active implementation of these CPGs and their recommendations is necessary [19]. One of the first steps for implementation is to identify the evidence-practice gaps in the management of shoulder pain by family physicians and physiotherapists [20, 21]. In the last decade, evidence-practice gaps in shoulder pain management have been studied in family physicians [22–24] and physiotherapists [24–28] of various countries. However, no recent studies compared shoulder pain management between family physicians and physiotherapists and no recent studies evaluating shoulder pain primary care management were conducted in Canada.

Using a survey design, the overall aim of this study was to describe knowledge and confidence of family physicians and physiotherapists in the province of Quebec in diagnosing and managing four common shoulder disorders. The study also aims to evaluate appropriateness of care by comparing the indicated management by family physicians and physiotherapists with recommendations from high-quality CPGs [6, 13–18] and to compare management and confidence between family physicians and physiotherapists in taking care of patients with shoulder pain.

## Methods

### Study design

This descriptive study used a cross-sectional survey design that follows the guidelines for reporting survey-based research [29]. The study was approved by the Health Research Ethics Committee of the CIUSSS-de-l’Est-de-l’Île de Montréal (2021–2224) in Montreal, Quebec, Canada.

### Study population

The survey was sent to physiotherapists via the email list and social media accounts of the *Ordre professionnel de la physiothérapie du Québec* (OPPQ) (Quebec’s Physiotherapy Professional College) and to family physicians through the *Réseau-1 Quebec* newsletter. *Réseau-1 Quebec* is a primary care knowledge and research network for clinicians and researchers aiming at facilitating research and uptake of evidence in primary care. The link of the survey was also sent via the email lists of selected physiotherapy clinics and university family medicine groups where the research team has ongoing collaborations (*n* = 6). The survey was active from February 18^th^ to June 11^th^, 2021. Based on our previous surveys sent out via professional associations, we expected a participation rate of 3 to 5% [30, 31]. Considering that approximately 5200 physiotherapists were licensed in 2020[32], we expected that 156 to 260 physiotherapists would answer the survey. Since approximatively 4700 family physicians are working in family medicine groups in the province of Quebec[32], we expected that 141 to 235 family physicians would answer the survey, [30, 31].

### Survey development

Socio-demographic and clinical characteristics of the respondents were collected. The survey included four clinical vignettes presenting patients with the following shoulder pain conditions: rotator cuff (RC) tendinopathy, acute full-thickness (FT) RC tear, adhesive capsulitis or traumatic anterior glenohumeral instability. The vignettes were selected and adapted by our research team based on published survey-based studies on shoulder pain management conducted in the United States, Australia and the United Kingdom [22, 23, 33]. The four vignettes are presented in Additional file 1. Each vignette was followed by a questionnaire on initial shoulder pain management and treatment that the respondent would recommend. This section of the survey was developed by the research team based on previous studies evaluating family physicians and physiotherapists’ evidence-practice gap for general MSK disorders and shoulder pain management [22, 23, 33–36]. We also questioned family physicians and physiotherapists on their overall confidence level (not confident at all, slightly confident, somewhat confident, fairly confident, completely confident) in making an appropriate diagnosis, selecting appropriate investigations, adequately referring patients to a medical MSK specialist, and selecting appropriate treatments when they are managing shoulder pain in their everyday practice.

#### Initial shoulder pain management

For each clinical vignette, respondents had to indicate: 1- the associated shoulder pain diagnosis (RC tendinopathy, acute FT RC tear, chronic RC tear, glenohumeral osteoarthritis, glenohumeral instability, adhesive capsulitis, acromioclavicular disorders, shoulder pain referred from the neck or other) 2- if they would recommend any diagnostic imaging test (blood tests, X-ray, diagnostic MSK ultrasound, magnetic resonance imaging [MRI], magnetic resonance arthrography [MRA] or any other test) and for what reason they would recommend those tests (to confirm diagnosis, to exclude other diagnoses, to guide treatment or to decide on a specialist referral) and 3- if they would refer the patient to a medical MSK specialist (orthopaedic surgeon, rheumatologist, physiatrist, sport physician or any other medical specialist) at the initial consultation with the patient.

#### Medical treatments

Family physicians and physiotherapists had to select, if any, medical treatments they would prescribe or recommend (oral non-steroidal anti-inflammatory drugs [NSAIDs], acetaminophen, opioid medication, corticosteroid infiltration, arthrographic distension or other).

#### Rehabilitation treatments

Family physicians had to indicate which rehabilitation treatments they would prescribe (physiotherapy referral, advice and education, home exercise program or other). Physiotherapists also had to indicate rehabilitation interventions they would provide, but possible answers detailed more specific interventions offered by physiotherapists. For each proposed rehabilitation intervention (education, active mobility exercises, passive mobility exercises, strengthening exercises, motor control exercises, manual therapy, thermotherapy, electrotherapy), physiotherapists had to indicate the priority of the intervention on a 6-point scale, 0 representing an intervention not to use and 5 representing an intervention that is extremely important to use.

### Appropriateness of care

To determine the appropriateness of the physiotherapists’ and family physicians’ care offered for all vignettes, recommended management was based on previous survey studies using the same clinical vignettes [22, 23, 33] as well as on the evidence-based recommendations of a recent CPG covering the initial management, medical and rehabilitation treatments of RC disorders in the context of the province of Quebec developed by our team [17]. We also systematically reviewed the literature to identify CPGs covering the management of RC disorders and other common shoulder disorders such as adhesive capsulitis and glenohumeral instability (PROSPERO: CRD42022325614) [37]. In the systematic review, we used a combination of keywords and Medical Subject Headings (MesH) terms including “shoulder”, “rotator cuff”, “adhesive capsulitis”, “GH osteoarthritis”, “GH instability”, “acromioclavicular” and “guidelines”. The search of CPGs published between 2008 and August 2022 was performed in four databases (Medline, Embase, Physiotherapy Evidence Database [PEDro], Google Scholar) and in international CPG databases. In the systematic review process, two reviewers assessed the methodological quality of the CPGs with the AGREE (Appraisal of Guidelines Research and Evaluation) II checklist and extracted the recommendations [38]. We identified five high-quality CPGs covering the management of RC disorders [6, 13, 15, 17, 18], two high-quality CPGs covering the management and indications for diagnosis imaging of adhesive capsulitis [13, 14] and two high-quality CPGs including indications for diagnosis imaging of traumatic anterior glenohumeral instability [6, 13]. We considered which CPGs were of high quality by using a frequently reported method in which domain three and at least two other domains of the AGREE II checklist had a score equal or over 60% [39]. We also included the CPG covering the medical and rehabilitation management of traumatic anterior glenohumeral instability with the highest overall score, since according to our assessment, no high quality CPGs covered the management of this condition [16]. Recommendations on shoulder pain management that were used to asses appropriateness of care are summarized in Table 1. Appropriateness of care was determined by one author (VL) and revised by a second author (FD).

**Table 1:** Recommendations from selected high quality clinical practice guidelines on the management of shoulder disorders used to assess appropriateness of care offered by physiotherapists and family physicians

Shoulder disorders	Initial management	Medical treatment	Rehabilitation treatment
Rotator cuff tendinopahy	X-rays are the first line examination for shoulder pain [6]/X-rays are not initially indicated in the initial management of RC tendinopathy [13, 17] (Conflicting recommendations^a^) US or MRI are not recommended in the initial management of RC tendinopathy [17] A referral to a medical specialist is not recommended in the initial management of RC tendinopathy [17]	Acetaminophen is recommended for pain relief [17]. Oral NSAIDs may be useful for short term pain relief [17]. Corticosteroids injections are not recommended as first line treatment to reduce pain and improve function, but may be useful to reduce pain and improve short term function [17]. Opioids are not recommended as first line pharmalogical treatment to reduce pain in disability. Opioids may be useful to reduce short term pain in adults that present severe pain and disability refractory to other analgesic modalities [17].	An active and functional rehabilitation program is recommended as an initial modality to reduce pain and improve function (Mobility, motor control, strengthening, endurance, education). It is recommended to prioritize active mobilization to passive modalities to reduce pain and improve function [17]. Manual therapy can be useful provided alone or with other modalities such as exercises to reduce pain and improve function [17]. Ultrasound, laser and extracorporeal shockwave treatment are not recommended to reduce pain and improve function [17]. Insufficient evidence to formulate recommendations for taping, TENS, iontophoresis, pulsed electromagnetic field, interferential current [17].
Acute full-thickness rotator cuff tear	X-ray, US or MRI are recommended in the presence of a suspected FT RC tear. US should be prioritized, when possible, because of lower costs and diagnostic properties similar to MRI [17]. A referral to a medical specialist is recommended in the suspicion of FT RC tear confirmed by an imaging test in the presence of important pain and/or muscular weakness and/or a significant activity limitation [17, 18].	Acetaminophen may be useful for short term pain relief [17]. Oral NSAIDs may be useful for short term pain relief [17]. Corticosteroids injections are not recommended as first line treatment to reduce pain and improve function, but may be useful to reduce pain and improve short term function [17]. Opioids are not recommended as first line pharmacological treatment to reduce pain in disability. May be useful to reduce short term pain in adults that present severe pain and disability refractory to other analgesic modalities [17].	An active rehabilitation program is recommended as an initial modality. Active modalities such as exercises should be included as early as possible [17]. Insufficient evidence to formulate recommendations for iontophoresis, pulsed electromagnetic field, interferential current [17].
Adhesive capsulitis	X-rays are not initially indicated [13]. Referral to a medical specialist for a surgical opinion: No recommendation regarding adding manipulation under anesthesia [14].	No recommendation from CPGs on use of acetaminophen NSAIDs is recommended in combination with outpatient physiotherapy (with passive mobilizations) [14]. An intra-articular steroid injection is recommended, preferably in combination with outpatient physiotherapy (with passive mobilizations) [14]. No recommendation from CPGs on opioids use	Outpatient physiotherapy (with passive mobilizations) with home exercises is recommended [14]. For stiffness-predominant frozen shoulder, probably use high grade mobilizations in preference to low grade mobilizations [14]. Thermotherapy is not recommended [14].
Traumatic anterior glenohumeral instability	X-rays are indicated [6, 13]. Advanced diagnostic imaging (MRI, MRA) is recommended [6, 13]. Referral to a medical specialist for a surgical opinion: Arthroscopic or open surgery is recommended for acute first anterior shoulder dislocation, particularly in patient under age 27 [16].	Acetaminophen is recommended [16]. Oral NSAIDs are recommended [16]. No recommendation from CPGs on corticosteroid infiltration Judicious short-term use of opioids is recommended for pain management for select patients with acute moderate to severe pain associated with shoulder dislocation. Opioids are not recommended for subacute or chronic pain [16].	Exercises are recommended [16]. Thermotherapy is recommended [16]. No recommendation for manual therapy, therapeutic ultrasound, TENS, iontophoresis, laser [16]. Taping, pulsed electromagnetic field and interferential current are not recommended [16].

*RC* Rotator cuff, *FT* Full thickness, *US* Ultrasound, *MRI* Magnetic resonance imaging, *MRA* Magnetic resonance arthrography, *NSAIDs* Non-steroidal anti-inflammatory drugs, *TENS* Transcutaneous electrical nerve stimulation, *CPG* Clinical practice guideline

^a^One CPG recommend an X-ray in the initial management of RC tendinopathy and two CPGs do not recommend X-ray in the initial management of RC tendinopathy

### Data analysis

Descriptive statistics were summarized for demographics and clinical characteristics of respondents and for results on shoulder pain management. For analysis purposes, we recoded results regarding the level of confidence into not confident (not confident at all or slightly confident), moderately confident (somewhat confident) and highly confident (fairly confident or completely confident). We also recoded the level of priority of rehabilitation interventions selected by physiotherapists as not a priority or low priority (0 and 1 on the 6-point scale), moderate priority (2 and 3 on the 6-point scale) and high priority (4 and 5 on the 6-point scale). Results regarding the initial management of shoulder disorders, medical treatments selected and confidence in shoulder pain management were compared between physiotherapists and family physicians using Fisher’s exact tests. We used Excel Version 16 to summarize data and RStudio Version 1.4.1106 for all statistical analysis. The alpha level was set at 0.05.

## Results

Two hundred-twenty physiotherapists initiated the survey, 175 completed at least the first vignette and 146 completed the entire survey. Ninety-three family physicians started the survey, and 76 completed the first vignette. Seventy-four family physicians completed the entire survey. Thus, the response rate for completing at least one vignette was 3% for the physiotherapists and 2% for the family physicians.

### Demographic and clinical characteristics of participants

Socio-demographic and clinical characteristics of respondents are presented in Table 2. More than 71% of the physiotherapists and family physicians were women. Most physiotherapists (62%) and family physicians (65%) had ten years of experience or less. Seventy percent of physiotherapists were working in private practice and 93% of family physicians practiced in a public setting, mostly in family medicine groups (96%).

**Table 2:** Socio-demographic and clinical characteristics of the participants

	PT (*n* = 175)		FP (*n* = 76)	
	n	(%)	n	(%)
Gender				
Women	127	73	54	71
Men	48	27	22	29
Age (years)				
18–24	11	6	0	0.0
25–34	96	55	34	45
35–44	46	26	20	26
45–54	18	10	12	16
55–64	4	2.3	8	11
65 +	0	0.0	2	3
Work Experience (years)				
0 to 5	70	40	39	51
6 to 10	38	22	10	13
11 to 15	23	13	3	4
16 to 20	23	13	5	7
21 to 25	10	6	7	9
25 +	11	6	12	16
Sector of practice				
Private	122	70	1	1
Public	36	21	71	93
Private and public	15	9	3	4
Other	2	1	1	1
Most common type of patients managed				
Pediatric	2	1	3	4
Adult	161	92	59	78
Geriatric	11	6	13	17
Not applicable	1	0.6	1	1
Percentage of patients treated for MSK disorders				
1–25	7	4	47	62
26–50	11	6	20	26
51–75	26	15	7	9
76–100	129	74	2	3
Not applicable	2	1	0	0.0
Percentage of patients treated for shoulder pain				
0	1	0.6	0	0.0
1–25	81	46	68	90
26–50	76	43	5	7
51–75	14	8	1	1
76–100	1	0.6	2	3
Not applicable	2	1	0	0.0
Work setting^a^				
Private clinic	135	77	2	3
Hospital	36	21	37	49
Readaptation center	10	6	0	0.0
Family medicine Group	3	2	73	96
Home care	12	7	17	22
Long term care residence	2	1	16	21
Research center	4	2	0	0.0
Other	8	5	5	7
Continuing education on MSK disorders				
Yes	149	85	29	38
No	26	15	47	62
Types of PT continuing education^a^				
Manual therapy	123	70		
Osteopathic approach	11	6		
Mckenzie approach	46	26		
Chronic pain treatment	39	22		
Postural approach	15	9		
Sports physiotherapy	37	21		
Motor control	5	2.9		
Shoulder specific courses	18	10.3		
Dry needling	9	5.1		
Other	11	6.3		

*PT* Physiotherapist, *FP* Family physician, *MSK* Musculoskeletal

^a^Respondents could select multiple answers. The total of answers could exceed 100%

### Confidence of physiotherapists and family physicians in shoulder pain management

When comparing the confidence level of family physicians and physiotherapists in shoulder pain management (Table 3), there were statistically significant differences (*p* < 0.001) favoring physiotherapists in confidence in making an appropriate diagnosis and selecting adequate treatments. More physiotherapists reported being highly confident for these type of management (respectively 64% and 80%) compared to the majority of family physicians that reported being only moderately confident (respectively 55% and 50%). No significant differences were observed between providers for selecting appropriate investigations and adequately referring patients to medical MSK specialists.

**Table 3:** Confidence level of physiotherapists and family physicians in shoulder pain management

	PT (*n* = 146)		FP (*n* = 74)		
*Confidence in…*	n	(%)	n	(%)	*p*-value ^a^
…making an appropriate diagnosis					
Highly confident	93	64	22	30	< 0.001*
Moderatly confident	49	34	41	55	
Not confident	4	3	11	15	
…selecting appropriate investigations					
Highly confident	70	48	31	42	0.32
Moderatly confident	62	43	39	53	
Not confident	14	10	4	5	
…adequately referring to medical MSK specialists					
Highly confident	77	53	31	42	0.31
Moderatly confident	57	39	35	47	
Not confident	12	8	8	11	
…selecting appropriate treatments					
Highly confident	118	81	32	43	< 0.001*
Moderatly confident	25	17	37	50	
Not confident	3	2	5	7	

*PT* Physiotherapists, *FP* Family physicians, *MSK* Musculoskeletal

^*^p < 0.05

^a^ Fishers tests were used to compare family physicians and physiotherapists

### Diagnosis and initial shoulder pain management

Selected diagnosis, indication for investigations and indication for medical MSK specialist referrals by family physicians and physiotherapists are presented in Table 4.

**Table 4:** Diagnosis and initial management of shoulder pain indicated by physiotherapists and family physician

	RC tendinopathy					Acute full-thickness RC tear					Adhesive capsulitis					Traumatic glenohumeral anterior instability				
	PT (*n* = 175)		FP (*n* = 76)		*p*-value	PT (*n* = 161)		FP (*n* = 74)		*p*-value	PT (*n* = 149)		FP (*n* = 74)		*p*-value	PT (*n* = 147)		FP (*n* = 74)		*p*-value
	n	(%)	n	(%)		N	(%)	n	(%)		n	(%)	n	(%)		n	(%)	n	(%)	
Adequate diagnosis																				
Yes	**140**	**80**	**64**	**84**	0.54	**144**	**89**	**71**	**96**	0.16	**147**	**99**	**70**	**95**	0.10	**145**	**99**	**74**	**100**	0.55
No	35	20	12	16		17	11	3	4		2	1	4	5		2	1	0	0.0	
Recommendation of investigation																				
Yes	22	13	23	30	0.001^a^	**107**	**67**	**65**	**88**	0.001^a^	33	22	38	51	0.02^a^	**60**	**41**	**56**	**76**	< 0.001^a^
None	**153**	**87**	**53**	**70**		54	34	9	12		**116**	**78**	**36**	**49**		87	59	18	24	
Type of recommended investigation^b^																				
	PT (*n* = 22)		FP (*n* = 23)			PT (*n* = 107)		FP (*n* = 65)			PT (*n* = 34)		FP (*n* = 38)			PT (*n* = 60)		(*n* = 56)		
Blood tests	1	4	1	4	1.00	0	0.0	0	0.0	1.00	1	3	7	18	0.06	0	0.0	1	1	0.48
X-Rays	13	59	21	91	0.02^a^	**4**	**3**	**4**	**5**	0.48	17	50	31	82	0.006^a^	**23**	**16**	**41**	**55**	0.01^a^
Diagnostic MSK US	10	46	5	22	0.12	**79**	**49**	**44**	**60**	0.39	13	38	8	21	0.13	6	4	9	12	0.41
MRI	1	5	0	0.0	0.49	**47**	**29**	**25**	**34**	0.53	2	6	1	3	0.60	**19**	**13**	**11**	**15**	0.20
MRA	1	5	0	0.0	0.49	10	6	2	3	0.14	4	12	0	0.0	0.05	**21**	**14**	**11**	**15**	0.10
Other	1	5	0	0.0		0	0.0	0	0.0		0	0.0	1	3		0	0.0	1	1	
Reason(s) for recommending an investigation^b^																				
	PT (*n* = 22)		FP (*n* = 23)			PT (*n* = 107)		FP (*n* = 65)			PT (*n* = 34)		FP (*n* = 38)			PT (*n* = 60)		(*n* = 56)		
To confirm diagnosis	8	36	7	30	0.75	**81**	**76**	**55**	**74**	0.23	13	38	8	21	0.12	**27**	**45**	**22**	**39**	0.57
To exclude another diagnosis	14	64	17	74	0.53	24	22	11	15	0.43	**15**	**44**	**33**	**87**	< 0.001^a^	33	55	38	68	0.18
To guide treatment	8	36	4	17	0.18	32	30	23	31	0.50	10	29	10	26	0.80	12	20	15	27	0.51
To refer the patient to a medical MSK specialist	3	14	0	0.0	0.11	**74**	**69**	**39**	**53**	0.25	7	21	2	5	0.07	**29**	**48**	**25**	**45**	0.83
Other	1	5	0	0.0		4	4	3	4		1	3	3	8		2	3	3	5	
Recommendation of referral to a medical MSK specialist^b^																				
	PT (*n* = 175)		FP (*n* = 76)			PT (*n* = 159)		FP (*n* = 74)			PT (*n* = 149)		FP (*n* = 74)			PT (*n* = 147)		FP (*n* = 74)		
None	**173**	**99**	**75**	**99**	1.00	88	55	53	72	0.01^a^	**120**	**81**	**69**	**93**	0.02^a^	99	67	37	50	0.02^a^
Orthopaedic surgeon	0	0.0	0	0.0	1.00	**59**	**37**	**20**	**27**	0.18	12	8	0	0.0	0.01^a^	**44**	**30**	**34**	**46**	0.03^a^
Other^c^	3	2	1	1	1.00	12	8	1	1	0.06	19	13	5	7	0.25	15	10	5	7	0.46

Proportion indicated in bold represent management that is recommended

*RC* Rotator cuff, *FP* Family physicians, *PT* Physiotherapists, *MSK* Musculoskeletal, *US* Ultrasound, *MRI* Magnetic resonance imaging, *MRA* Magnetic resonance arthrography

^a^Statistically significant difference between PT and FP as determined by a Fisher’s test (*p* < 0.05)

^b^Participants could select more than one option. The total of answer can exceed 100%

^c^Rheumatologist, physiatrist, sport physician or other medical specialist

#### RC tendinopathy vignette

The RC tendinopathy vignette presented a healthy 77-year-old woman with non-traumatic shoulder pain in the last six weeks and normal range of motion, but with pain on mid-range active abduction (Additional file 1). The most probable diagnosis for this patient was a RC tendinopathy, but a chronic RC tear would also be a plausible diagnosis because of the patient’s age. Eighty percent of physiotherapists and 84% of family physicians selected either RC tendinopathy or chronic RC tear as a diagnosis and were considered as having selected the correct diagnosis for the patient. GH osteoarthritis was not considered an adequate diagnosis since the patient presented with complete shoulder range of movement. There was no statistically significant difference between physiotherapists and family physicians (*p*= 0.54) in selecting the adequate diagnosis. CPGs do not recommend any medical or diagnostic imaging tests in the initial management of RC tendinopathy [13, 17]. Significantly more family physicians (30%) than physiotherapists (13%) recommended a medical test or a diagnostic imaging (*p*= 0.001). Most clinicians indicated that they would recommend a diagnostic imaging test to exclude another diagnosis. Almost all respondents (> 99%) did not recommend referring the patient with a suspected RC tendinopathy to a medical specialist, which is considered appropriate care [17].

#### Acute FT RC tear vignette

The second vignette presented a 45 year-old worker with a traumatic onset of shoulder pain that happened two weeks prior with inability to work and to raise his arm above shoulder level (Additional file 1). There was no significant difference (*p*= 0.16) in the proportion of respondents making the appropriate diagnosis of an acute FT RC tear (physiotherapists (89%) and family physicians (96%). In the case of a suspected acute FT RC tear, especially in a young worker with important disability, a diagnostic imaging is recommended [17]. Significantly more family physicians than physiotherapists adequately recommended diagnostic imaging for this case (88% vs 67%, *p* = 0.001). Most clinicians indicated that they would recommend a MSK diagnostic ultrasound (PT: 49%, FP: 60%) or an MRI (PT: 29%, FP: 34%) with the aim to confirm the diagnosis (PT: 76%, FP: 74%). Fifty-five percent of physiotherapists and 72% of family physicians (*p*= 0.01) would not initially recommend referring the patient to a medical MSK specialist in the initial management of an acute FT RC tear, although a rapid referral to a medical MSK specialist is recommended [17].

#### Adhesive capsulitis vignette

Most physiotherapists (99%) and family physicians (95%) adequately selected the adhesive capsulitis (*p* = 0.10) diagnosis for the vignette presenting a 50-year-old woman with a 3-week history of shoulder pain without trauma and progressive limitations of range of motion (Additional file 1). More than half of family physicians (51%) indicated that they would prescribe an imaging test, which is significantly higher (*p*= 0.02) than for physiotherapists (22%). According to recommendations, an x-ray or any other diagnostic imaging is not necessary in suspected cases of adhesive capsulitis [13]. Most family physicians (87%) and physiotherapists (41%) (*p* < 0.001) recommended an imaging test to exclude other diagnoses. Most physiotherapists (81%) and family physicians (93%) (*p*= 0.02) did not recommend referring the patient to a medical MSK specialist (orthopaedic surgeon, rheumatologist or sport physician), which is in line with recommendations from CPGs [14].

#### Traumatic anterior glenohumeral instability

The traumatic anterior glenohumeral instability vignette presented a 21-year-old woman that suffered a traumatic dislocation 6-weeks prior and following that initial trauma had had episodes of shoulder subluxation (Additional file 1). All family physicians and 99% of physiotherapists (*p* = 0.55) adequately selected glenohumeral instability as the correct diagnosis. One CPG states that an x-ray and advanced imaging such as MRI or MRA are indicated in such cases [13] and another CPG also recommend reference to orthopedic surgeon for a first time traumatic dislocation, particularly in patients 27 years old or younger [16]. Significantly more family physicians (76%) than physiotherapists (41%) (*p* < 0.001) adequately indicated that they would recommend an investigation for that patient. Only 30% of physiotherapists and 46% of family physicians indicated that they would refer the patient to an orthopaedic surgeon (*p* = 0.02).

### Medical care

**Fig. 1:**
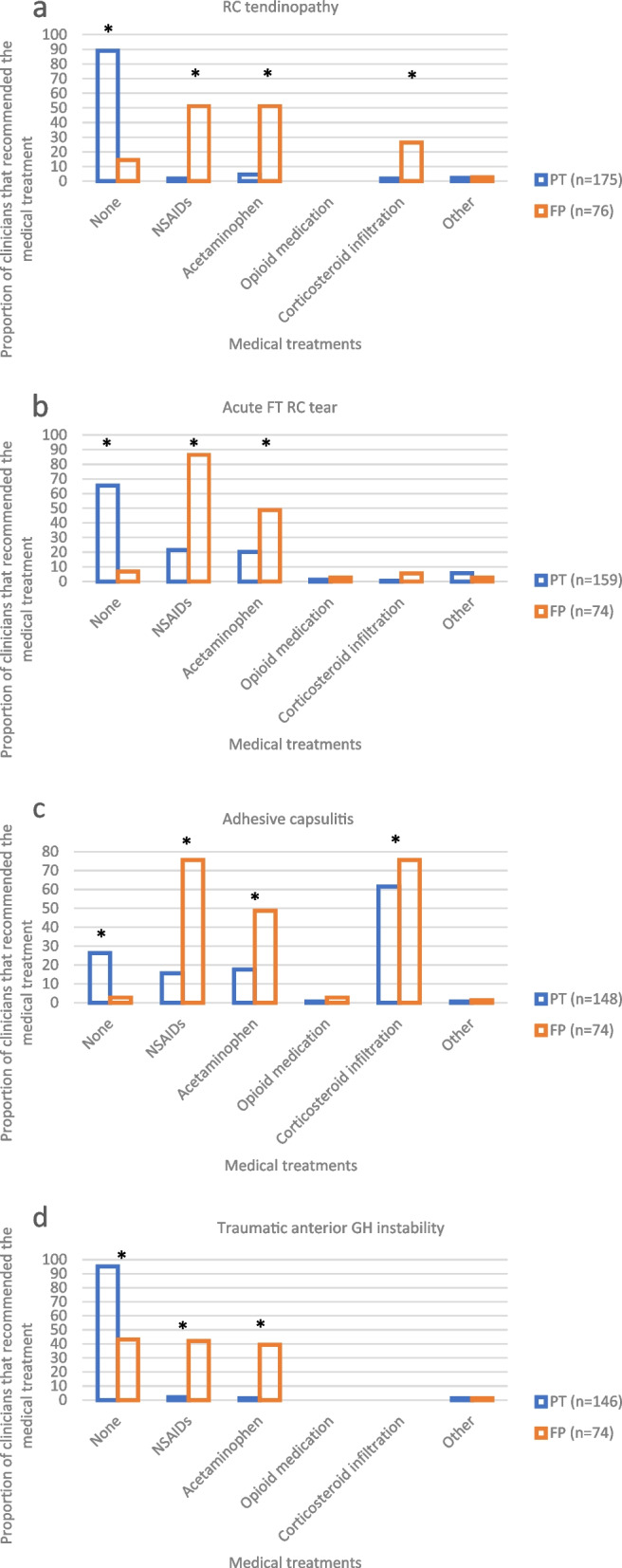
a-d Medical treatment selected by family physicians and physiotherapists. RC: rotator cuff, FT: Full thickness, GH: Glenohumeral instability, PT: Physiotherapists, FP: Family physicians, NSAIDs: Non-steroidal anti-inflammatory drugs. *Statistically significant difference between PT and FP as determined by a Fisher’s test (*p* < 0.05)

Medical treatments selected by family physicians and physiotherapists in the management of shoulder pain patients described in the clinical vignettes are presented in Fig. 1 (a to d).

#### RC tendinopathy vignette

Acetaminophen and oral NSAIDs may be useful, while corticosteroid infiltrations and opioids are not recommended as first line treatment options in the management of RC tendinopathy [17]. Most physiotherapists (89%) did not recommend any medical treatments, compared to only 15% of family physicians (*p*< 0.001). Respectively 42% and 51% of family physicians would prescribe acetaminophen and oral NSAIDs. No family physicians or physiotherapists prescribed opioids, but 26% of family physicians indicated that they would prescribe a corticosteroid infiltration, which is not recommended [17].

#### Acute FT RC tear vignette

Recommendations for the non-surgical medical management of an acute FT RC tear are similar to medical treatments recommended for RC tendinopathy [17]. Approximatively 20% of physiotherapists recommended oral NSAIDs and acetaminophen, while 76% of family physicians recommended NSAIDs and 48.6% of them recommended acetaminophen (*p* < 0.001). Very few family physicians and physiotherapists recommended any opioid medication (PT: 1%, FP: 3%, *p* = 0.59) or a corticosteroid infiltration (PT: 0.6%, FP: 5%, *p* = 0.04).

#### Adhesive capsulitis vignette

Appropriate care for adhesive capsulitis should include an intra-articular corticosteroid or an arthrographic distension, preferably combined with physiotherapy treatments that include shoulder range of motion exercises [14]. Oral NSAIDs may also be prescribed in combination with physiotherapy treatments including passive mobilization [14]. Corticosteroid infiltrations or arthrographic distension were recommended by significantly more family physicians (76%) than physiotherapists (62%) (*p* = 0.04). Most family physicians (76%) recommended oral NSAIDs, compared to only 16% of physiotherapists (*p* < 0.001).

#### Traumatic anterior glenohumeral instability vignette

Acetaminophen and NSAIDs may be recommended in the management of pain related glenohumeral instability but was not considered to be necessary in this vignette since the woman only suffered minimal discomfort at this stage. Respectively 42% and 39% of family physicians did prescribe oral NSAIDs or acetaminophen, compared to 2% and 1% of physiotherapists (*p* < 0.001). No family physicians or physiotherapists recommended opioid medication in the management of this case.

### Rehabilitation care

**Fig. 2:**
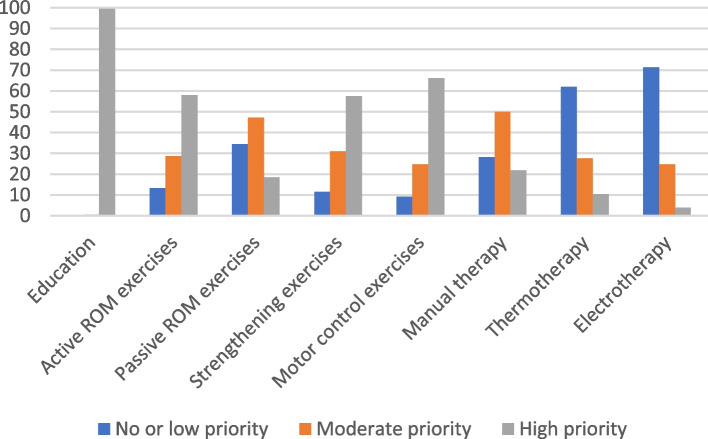
Level of priority of treatment indicated by physiotherapists for RC tendinopathy. RC: Rotator cuff, ROM: Range of motion

**Fig. 3:**
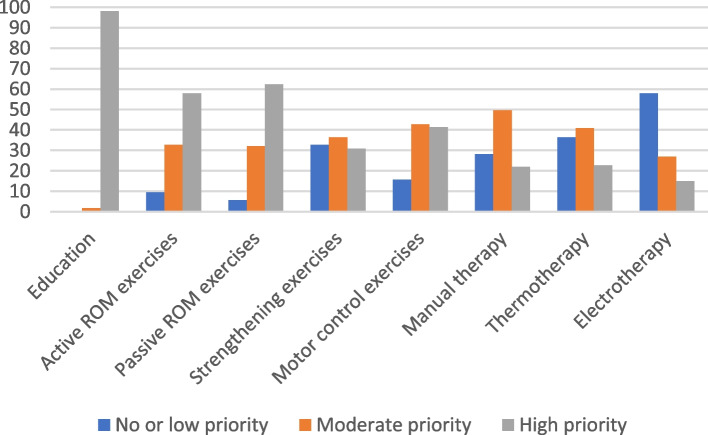
Level of priority of treatment indicated by physiotherapists for acute FT RC tear. FT: Full thickness, RC: Rotator cuff, ROM: Range of motion

**Fig. 4:**
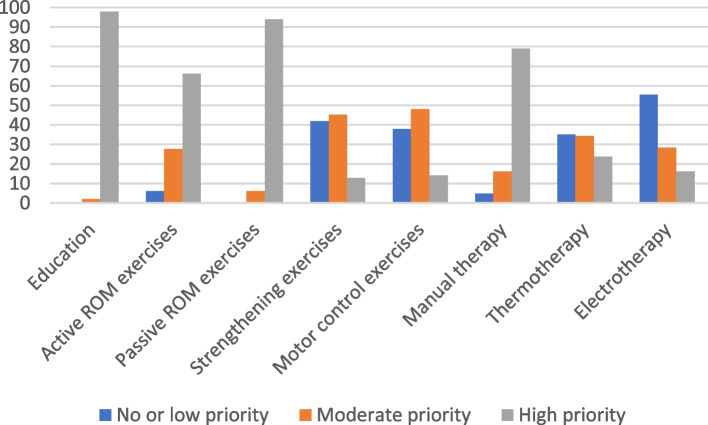
Level of priority of treatment indicated by physiotherapists for adhesive capsulitis. ROM: Range of motion

**Fig. 5:**
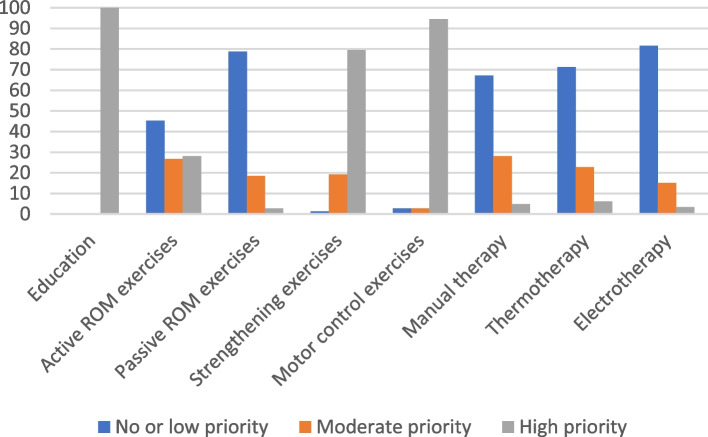
Level of priority of treatment indicated by physiotherapists for traumatic anterior GH instability. GH: Glenohumeral. ROM: Range of motion

The rehabilitation treatments prescribed by family physicians are presented in Table 5. The rehabilitation treatments recommended by physiotherapists are presented in Figs. 2, 3, 4 and 5.

**Table 5:** Rehabilitation treatments recommended by family physicians

	RC tendinopathy		Acute FT RC tear		Adhesive capsulitis		Traumatic anterior GH instability	
	*n* = 76		*n* = 74		*n* = 74		*n* = 74	
	n	%	n	%	n	%	n	%
**Rehabilitation**^a^								
Reference for physiotherapy	72	95	63	85	70	95	68	92
Advice and education	63	83	58	78	56	76	66	89
Home exercise program	49	65	37	50	48	65	41	55

RC: Rotator cuff, FT: Full thickness, GH: Glenohumeral

^a^Participants could select more than one option. The total of answer can exceed 100%

#### RC tendinopathy vignette

For RC tendinopathy, an active rehabilitation program including education and exercise is recommended [17, 40, 41]. Rehabilitation interventions are often provided by physiotherapists, thus most family physicians (95%) adequately recommended referring the patient for physiotherapy treatments. More than four out five family physicians (83%) indicated that they would provide advice and education to the patient, but fewer respondents (65%) indicated that they would give the patient a home exercise program.

Regarding rehabilitation treatments selected by physiotherapists, education was indicated as a high priority by 99% of physiotherapists. Active mobility exercises and strengthening exercises were indicated as a high priority by 58% of physiotherapists and motor control exercises by 66% of physiotherapists. These percentages are considered low for exercise interventions, since an active rehabilitation program should include these type of exercises [17]. Manual therapy, which can be useful to reduce pain and improve function [17], was indicated as being of moderate priority by 50% of physiotherapists and as a high priority by 22% of physiotherapists. Therapeutic ultrasound, laser and extracorporeal shockwave are not recommended in the treatment of RC tendinopathy and there is a lack of evidence to support the use of TENS, iontophoresis, pulsed electromagnetic field or interferential current [17]. There are no recommendation from CPGs on thermotherapy, but CPGs indicate that active modalities should be prioritized [17]. Thus, all passive physical modalities should not be a priority in the rehabilitation of this patient. Electrotherapeutic and thermotherapy were not a priority or a low priority according to respectively 71% and 62% of physiotherapists.

#### Acute FT RC tear vignette

Despite an acute FT RC tear requiring an early referral for diagnostic imaging and to a medical MSK specialist, it is recommended to initiate an active rehabilitation treatment with exercises as early as possible [17]. Eighty five percent of family physicians indicated that they would adequately refer the patient for physiotherapy treatments, but only half of the family physicians respondents would give this patient a home exercise program. Most family physicians (78%) would educate the patient that suffered an acute FT RC tear.

Patients’ education was also a high priority for 98% of physiotherapists. The rehabilitation modality that was considered as being of high priority by most physiotherapists (62%) was stretching and passive mobility exercises. Active mobility exercises (58%) and strengthening exercises (37%) were less prioritized, which is considered low value choices since active modalities should be included in the rehabilitation interventions [17].

#### Adhesive capsulitis vignette

Regarding the adhesive capsulitis vignette, 95% of family physicians would refer the patient for physiotherapy, which is recommended [14]. A home exercises program is also recommended and 65% of family physicians did indicate they would prescribe one. Three quarters of family physicians’ respondents would provide advice and education to the patient. Education (98%), stretching and passive mobility exercises (94%) as well as manual therapy (79%) were considered as a high priority by physiotherapists. These are considered appropriate interventions for this clinical vignette [14].

#### Traumatic anterior glenohumeral instability vignette

Based on CPG recommendations, exercises and thermotherapy are recommended interventions for glenohumeral instability [16]. However, the patient presented in the vignette did not suffer from pain, thus thermotherapy is not necessary. Most family physicians (92%) did recommend referring the patient for physiotherapy treatments and 89% would provide advice and education. However, only 55% of family physicians would give the patient a home exercise program.

Strengthening and motor control exercises were indicated as a high priority by respectively 80% and 95% of physiotherapists. All physiotherapists would educate the patient. According to 71% of physiotherapists, thermotherapy was not a priority or a low priority. Electrotherapy modalities such as pulsed electromagnetic field and interferential current are not recommended based on GPGs [16]. More than 80% of physiotherapists indicated that electrotherapy was not a priority or a low priority.

## Discussion

The aim of this survey study was to describe knowledge, confidence and appropriateness of care of family physicians and physiotherapists in the management of a recent onset of shoulder pain in four selected clinical vignettes and to determine the gap between clinical practice and recommendations from high-quality CPGs [6, 13–18]. Overall, there was variability between management indicated by family physicians and physiotherapists and both types of providers indicated referrals or treatments that did not correspond to recommended care.

### Confidence in managing shoulder pain

The vast majority of physiotherapists were highly confident in diagnosing and managing shoulder pain but only a minority of physicians were. Physiotherapists do have extensive training in the diagnosis and conservative management of shoulder pain. At least a third of physiotherapists’ training in Canada is in the management of MSK disorders [42], while around 3% of the Canadian undergraduate family physician curriculum is dedicated to MSK management [43]. This may explain the significant differences between confidence of family physicians and physiotherapists in selecting a diagnosis and treatments for shoulder pain patients. Moreover, in our sample, experience with treating shoulder pain was not equivalent between providers. Physiotherapists reported offering care to shoulder pain patients more frequently.

### Initial shoulder pain management

A very high proportion of family physicians and physiotherapists selected the appropriate diagnoses for the presented vignettes. These results are encouraging since family physicians in our study reported only being moderately confident in selecting a diagnosis and treatments for shoulder pain patients, while most physiotherapists reported being more confident. The family physicians’ performance here is comparable to a United Kingdom study in which 82% and 92% of respondents adequately identified a RC tendinopathy and an adhesive capsulitis, based on the same vignettes as those in our study [22]. However, these survey results may not represent clinical practice as shoulder pain history and signs and symptoms described in the clinical vignettes were relatively clear and uncomplicated [44]. Also, respondents from the survey may not be totally representative of the general population of family physicians and physiotherapists because participants may be more likely to have an interest in shoulder pain or MSK disorders management.

For all vignettes, family physicians recommended significantly more investigations (30 to 88%) than physiotherapists (13 to 67%). In RC tendinopathy or adhesive capsulitis vignettes, the performance of family physicians was not optimal as imaging tests are not recommended for the initial management of these two disorders. The proportions observed here are however lower than in studies conducted in the United Kingdom and Australia in which up to 82% of family physicians did recommend imaging for these shoulder cases [22, 23]. It has been reported that family physicians often overprescribe investigations because of the fear of missing a serious pathology [45]. The results from our survey study tend to confirm this since up to 87% of family physicians that ordered an imaging test, did so to exclude other pathologies.

Other studies looking at the ability of physiotherapists to adequately refer for diagnostic imaging report a referral rate of up to 31% for tendinopathy [27, 46] and 54% for adhesive capsulitis management [47], which is higher than in our study (13% and 22% respectively). Thus, in cases where diagnostic imaging is not recommended, physiotherapists here were more likely to manage shoulder pain as recommended by CPGs. It remains unclear if this behaviour is related to the scope of practice of physiotherapists in the province of Quebec where they do not autonomously prescribe imaging tests in most situations.

As for the management of acute FT RC tear and traumatic anterior glenohumeral instability, referring the patient for an investigation and a surgical opinion are recommended, but physiotherapists less often recommended such care. More family physicians appropriately managed these disorders. Physiotherapists may be more confident in the efficacy of non-surgical treatment [48], but only at a later stage would they recommend a diagnostic imaging if the patient’s condition is not improving. However, even though a rehabilitation program can be initiated for these pathologies [16, 17], standard of care is to refer these patients to a medical MSK specialist [16, 17]. Only 27 to 46% of family physicians and 30 to 37% of physiotherapists did initially refer the patient to an orthopaedic surgeon for a surgical opinion for acute FT RC tear and traumatic anterior glenohumeral instability. This result is concerning since delays in surgery are associated with poorer outcomes for these disorders [49–53]. The low levels of referral to medical MSK specialists in our study may be explained however by the administrative requirement of several orthopaedic departments in the Province of Quebec to include results of a diagnostic ultrasound or an MRI when referring the patient for a surgical consultation [54]. Since the survey questions were on shoulder pain management at the initial consultation, several respondents may have not indicated that they would refer the patient to a medical MSK specialist at that moment since they would wait confirmation of the diagnosis with the imaging results.

### Medical care

Most physiotherapists (65 to 95%) did not recommend any medical treatments for the four clinical vignettes, although oral NSAIDs and acetaminophen are recommended in painful shoulder conditions [14, 17]. Potential medical modalities that can reduce patients’ level of pain may be underused by physiotherapists, which may be explained by their scope of practice not allowing them to autonomously prescribe medication. However, physiotherapists are able to use effective active modalities and exercises to reduce pain and improve patients’ function [55]. In the four clinical vignettes, 39% to 87% of family physicians have indicated that they would prescribe oral NSAIDs or acetaminophen, as recommended by CPGs for short-term pain reduction [14, 17]. In the management of RC tendinopathy, there was a significantly higher reliance on the use of corticosteroids infiltrations by family physicians (27%), compared to physiotherapists (2%), even though this modality should not be used as an initial treatment for this pathology [17]. Using corticosteroids infiltrations in the management of RC tendinopathy by family physicians was also too often recommended in the study by Buchbinder et al. (24%) [23] and the study by Artus el al. (48%) [22]. The over-reliance on corticosteroids infiltration in the management of shoulder pain observed in our survey may be partially explained by the lack of other treatment options, such as poor access to free of charge physiotherapy in the Province of Québec or also patient preferences where some could prefer a quick reduction of symptoms [56, 57]. In the management of adhesive capsulitis however, an intra-articular corticosteroids infiltration is recommended, preferably combined with physiotherapy treatments including mobility exercises [14]. It appears that there is a need for education and other guideline implementation strategies [58] on the appropriate management of adhesive capsulitis among clinicians since 24% of family physicians and 38% of physiotherapists did not recommend an intra-articular corticosteroids infiltration or an arthrographic distension.

### Rehabilitation care

Regarding rehabilitation care, most family physicians reported that they would refer the patients presented in the vignettes to a physiotherapist (85–95%) and provide advice and education (78–89%), as recommended [14, 16, 17]. These proportions are higher than in previously published survey studies (57–77%) [22, 23]. However, in clinical practice, the actual referral rate to physiotherapists may be lower, because of the lack of access to publicly funded physiotherapy in our health care system, as already mentioned [56]. Several observational studies reporting on shoulder pain management by family physicians have demonstrated an actual referral rate to physiotherapy of 13–53% for patients with RC disorders or adhesive capsulitis [59–62]. Less family physicians indicated that they would give the patient a home exercise program (50–65%), despite this modality being an essential component of rehabilitation [63]. The development of self-management modules including exercises, general advice and education that are accessible to family physicians and patients could benefit patients’ condition but are not commonly available in clinical practice actually [64].

Almost all physiotherapists adequately indicated that they would provide advice and education to the patients, as recommended. In the management of RC tendinopathy, RC tear and glenohumeral instability, active exercises are recommended [16, 17]. However, for up to 43% of physiotherapists, strengthening and active mobility exercises were not indicated as being an important priority. These results are comparable to two studies that reported that only 54% to 67% would prescribe strengthening exercises in shoulder pain management [25, 26]. Passive modalities such as electrotherapy and thermotherapy are either not recommended or there are no recommendations in the management of most shoulder disorders [14, 16, 17]. Nonetheless, up to 65% and 45% of physiotherapists considered thermotherapy and electrotherapy as being a moderate or a high priority treatment. Active modalities should be prioritized in shoulder pain management since they can promote patients’ self-efficacy, and patients with higher levels of self-efficacy have a lower risk to develop chronic MSK pain [65, 66]. These results indicate a need to implement recommendations on evidence-based treatments that should be used for shoulder pain patients in clinical practice with strategies such as training and education, support of clinicians and development of relationships [4, 58].

### Strength and limitations of the current survey

This is the first study to evaluate and compare the management of shoulder pain between family physicians and physiotherapists since 2002 [67] and the only study to evaluate shoulder pain management in the Canadian context in the last twenty years [68]. The clinical vignettes were used in previous family physicians survey studies in other countries, which allowed us to compare management across settings [22, 23, 33]. To evaluate appropriateness of care, several high quality CPGs were selected using a systematic review process with a methodological quality assessment of their content [37, 38].

However, our study presents some limitations. Using high quality CPGs to assess quality of care of respondents involve that evidence from recent individual studies or systematic review may have not been considered in the evaluation. However, high quality CPGs are the highest level of evidence to help clinician manage patients’ conditions [69]. The principal limitation of our study is that the sample size of the study was relatively small. In the context of the COVID-19 pandemic, healthcare providers were very busy in their clinical practice, which may have limited their time to participate in this survey study. Another limitation of survey studies is that clinicians that decided to participate in the survey on shoulder pain management potentially have a greater interest in managing these disorders. Also, most respondents had less than 10 years of experience. Therefore, they may not be representative of all clinicians, physicians or physiotherapists. This bias may be especially present for family physicians in our study since our sample size is small and the management of family physicians was more concordant with recommendations than in previous studies using the same vignettes [22, 23]. Finally, survey studies with clinical vignettes do not entirely represent real clinical decision making and practice of family physicians or physiotherapists. Surveys may be easier to answer and does not take into account the clinical ability of providers in performing a valid questionnaire and physical examination. Yet using clinical vignettes has been reported as a valid and cost-effective option to evaluate health providers practice variations [70, 71].

## Conclusion

The vast majority of family physicians and physiotherapists were able to make adequate diagnoses and select appropriate treatments for shoulder pain. Based on the results from our survey, there is distinct needs to update the knowledge of family physicians and physiotherapists depending on the shoulder diagnosis so that their management can conform to evidence-based recommendations of high-quality CPGs. Education also needs to be targeted to the type of provider regarding the recommendation of diagnostic imaging tests, reference to medical MSK specialists and prescription of infiltration. Recommendations from CPGs on shoulder pain management regarding exercise prescription, and to avoid electrotherapy and thermotherapy were not always followed by physiotherapists. Actively implementing targeted recommendations from CPGs in clinical practice to help family physicians and physiotherapists adequately manage shoulder pain could optimize the use of health resources and ultimately improve patients’ care and health outcomes.

## Supplementary Information

**Additional file 1.** Vignettes adapted by our research team and presented to survey respondents.

## Data Availability

The datasets used and/or analyzed during the current study are available from the corresponding author on reasonable request.

## Abbreviations

PTs: Physiotherapists
FPs: Family physicians
RC: Rotator cuff
CPGs: Clinical practice guidelines
MSK: Musculoskeletal
OPPQ: Ordre professionnel de la physiothérapie du Québec
FT: Full-thickness
US: Ultrasound
MRI: Magnetic resonance imaging
MRA: Magnetic resonance arthrography
NSAIDs: Non-steroidal anti-inflammatory drugs
AGREE: Appraisal of Guidelines Research and Evaluation
