# Supplementary material for: Knowledge and appropriateness of care of family physicians and physiotherapists in the management of shoulder pain: a survey study in the province of Quebec, Canada
Source: BMC Prim Care. 2023 Feb 16;24:49. doi: 10.1186/s12875-023-01999-6 (PMC9933814; doi:10.1186/s12875-023-01999-6)
Supplement: Supplementary file 1 — Additional file 1. Vignettes adapted by our research team and presented to survey respondents. [file 12875_2023_1999_MOESM1_ESM.docx]

**Additional file 1:** Vignettes adapted by our research team and presented to survey respondents

**Vignette 1**

A 77-year-old woman, a retired bookkeeper living with her husband, presents with a 6-week history of discomfort in her right shoulder/deltoid region while sleeping, and difficulty doing her hair, putting on her coat, doing up her bra and reaching up to high shelves. There is no history of trauma. She has been previously well with no history of serious illness. A previous GP prescribed a 2-week course of NSAID, which didn’t provide any relief. On examination there is tenderness over the lateral aspect of the shoulder and pain on shoulder abduction in the mid-range but a normal range of movement. The remainder of the findings on physical examination are normal.

**Vignette 2**

A 45 year-old labourer sustained a work-related injury to his left shoulder two weeks ago. A 25kg door he was carrying slipped from his grip and he felt a sharp pain in his shoulder as he attempted to stop the door from falling. His foreman made him go to the hospital where x-rays of his shoulder were normal. Since then he reports that his pain is still present, but has improved. However, he has been unable to return to work because he cannot raise his arm above his chest height.

**Vignette 3**

A 50 year-old, right-hand-dominant female executive presents with a 3-week history of pain and progressive loss of motion of her left shoulder without history of trauma. The pain has been severe and interfering with sleep. On physical exam, a global loss of active and passive range of motion is noted with forward elevation to 90 degrees, internal rotation to the sacrum and external rotation to 10 degrees.

**Vignette 4**

A 21-year-old student presents with a complaint of left shoulder instability. Six weeks ago, she fell while going down the stairs and used her left arm to arrest her fall by holding on to the railing. She recovered from the initial pain and discomfort within 2 weeks of this incident, but she subsequently had 2 episodes of shoulder subluxation where she felt her shoulder “slipping in and out of place”. On clinical examination, there is no mobility restriction. The anterior elevation is 190 degrees bilaterally. External rotation at 90 degrees of abduction is 100 degrees bilaterally with apprehension on assessment of the left shoulder.
